# Ultrasonic Communication in Rats: Can Playback of 50-kHz Calls Induce Approach Behavior?

**DOI:** 10.1371/journal.pone.0001365

**Published:** 2007-12-26

**Authors:** Markus Wöhr, Rainer K. W. Schwarting

**Affiliations:** Experimental and Physiological Psychology, Philipps-University of Marburg, Marburg, Germany; University of Cambridge, United Kingdom

## Abstract

Rats emit distinct types of ultrasonic vocalizations, which differ depending on age, the subject's current state and environmental factors. Since it was shown that 50-kHz calls can serve as indices of the animal's positive subjective state, they have received increasing experimental attention, and have successfully been used to study neurobiological mechanisms of positive affect. However, it is likely that such calls do not only reflect a positive affective state, but that they also serve a communicative purpose. Actually, rats emit the highest rates of 50-kHz calls typically during social interactions, like reproductive behavior, juvenile play and tickling. Furthermore, it was recently shown that rats emit 50-kHz calls after separation from conspecifics. The aim of the present study was to test the communicative value of such 50-kHz calls. In a first experiment, conducted in juvenile rats situated singly on a radial maze apparatus, we showed that 50-kHz calls can induce behavioral activation and approach responses, which were selective to 50-kHz signals, since presentation of 22-kHz calls, considered to be aversive or threat signals, led to behavioral inhibition. In two other experiments, we used either natural 50-kHz calls, which had been previously recorded from other rats, or artificial sine wave stimuli, which were identical to these calls with respect to peak frequency, call length and temporal appearance. These signals were presented to either juvenile (Exp. 2) or adult (Exp. 3) male rats. Our data clearly show that 50-kHz signals can induce approach behavior, an effect, which was more pronounced in juvenile rats and which was not selective to natural calls, especially in adult rats. The recipient rats also emitted some 50-kHz calls in response to call presentation, but this effect was observed only in adult subjects. Together, our data show that 50-kHz calls can serve communicative purposes, namely as a social signal, which increases the likelihood of approach in the recipient conspecific.

## Introduction

Rats emit distinct types of ultrasonic vocalizations (USV), which differ depending on age, the subject's current state and environmental factors [1]–[3]. Rat pups typically exhibit USV in response to isolation from mother and litter [4]. Juvenile and adult rats, on the other hand, produce two different types of USV, which have been classified primarily on the basis of their sound frequency as low and high frequency vocalizations.

Low frequency vocalizations, often termed 22-kHz calls, are emitted when rats are exposed to predators [5], foot-shocks [6]–[10], during inter-male aggression [11], [12], drug withdrawal [13], [14], handling [15], and social isolation [16]. Remarkably, anxiolytic drugs can reduce such vocalizations [17]–[19]. Functionally, it was assumed that 22-kHz calls reflect a negative affective state akin anxiety and sadness [8], [9], and that they serve as alarm cries [5].

Conversely, high-frequency vocalizations, often termed 50-kHz calls, occur during or in anticipation of juvenile rough-and-tumble play [19], [20], mating [21]–[28], food consumption [29], electrical self-stimulation of the brain [29], [30], and addictive drugs [31]–[35]. Furthermore, rats also emit such calls when tickled by a skilled experimenter in a playful way [36]–[40], and rates of 50-kHz calls were found to be positively correlated with the rewarding value of tickle stimulation as measured by instrumental approach behavior [36], [37], [39]. Conversely, aversive stimuli including bright light [20], [37], predatory odors [37], the presence of foot shock cues [29] and drugs with aversive properties decrease levels of 50-kHz calls [41]. Based on such evidence, Panksepp and Burgdorf [40] suggested that 50-kHz calls might provide an archaic form of human laughter (“rat laughter”), which might serve as an index of the animal's subjective state [2]. Thereby, 50-kHz calls might provide a new and unique measure for analyzing natural reward circuits in the brain [29], [30], [42].

Recently, however, it was shown that 50-kHz calls can also occur in situations that are not necessarily pleasurable or even mildly aversive to rats. Thus, it was found that 50-kHz calls were emitted during short social isolation in the animal's own, or in a new soiled or fresh housing cage, irrespective of whether the animal's motivational status was high or low, i.e. irrespective of whether the animal was food-deprived or fed ad libitum [40], [43]. Also, during testing in an open field and an elevated plus maze 50-kHz calling was observed [43]. These findings are in line with observations of 50-kHz calls in various experimental controls, like naïve rats that were placed into a test arena containing fresh bedding [24], [44], or saline-injected rats in drug studies [33]–[35], [41]. Remarkably, the propensity to call differed dependent on the time-point of the last social contact, i.e. rats emitted 50-kHz calls primarily initially after separation from the cage mate [43]. Finally, it was found that not only the animal, which was isolated in a new housing cage emitted 50-kHz calls, but also the cage mate that remained alone in the home cage after the removal of the test rat [43]. These findings corroborated the idea that 50-kHz calls serve for communicative purposes, e.g. to (re)establish or keep contact.

A social function of rat USV was already confirmed successfully by performing playback studies in pups [45]–[47]. In adult rats, it was shown that the presentation of natural 22-kHz calls or 20-kHz sine wave tones can activate the fight/flight/freeze system [48]–[53]. However, little is known about the effects of 50-kHz calls on the behavior of the receiver. Schleidt [54] found that diverse artificial ultrasonic stimuli elicit Preyer's reflex, i.e. twitches of the auricles, in rats, and Thomas et al. [55] observed a suppression of instrumental bar pressing and bradycardia when artificial 50-kHz tones were presented. Apart from these early studies, responses to playback of high-frequency ultrasonic stimuli have been studied primarily within the sexual context. Here, changes in approach behavior [56], [57], proceptive behavior [22], [25], [27] and ultrasonic calling were observed [58]. Finally, two recent studies in non-sexual contexts obtained incongruent results. Burgdorf et al. [32] found that rats show instrumental behavior to receive playback of 50-kHz calls, whereas Endres et al. [59] did not find overt behavioral effects of 50-kHz playback.

The aim of the present study was to test the communicative value of 50-kHz calls by measuring overt and calling behavior during playback of such calls. As a testing environment, we used an unbaited radial-arm maze, since this apparatus had proven its usefulness in a previous experiment, where we had tested the behavioral effects of presenting pup 40-kHz calls to rat dams [47]. Here, it was hypothesized that presentations of 50-kHz calls induce locomotor activity and ultrasonic calling, whereas 22-kHz calls induce locomotor inhibition and a reduction in ultrasonic calling (Exp. 1). Furthermore, it was hypothesized that the 50-kHz call induced activation is stimulus-directed, i.e. that animals will approach the source of 50-kHz calls while calling themselves. Also, we assumed that the behavioral response is dependent on subject- and call-related features. Regarding subjects, we used juvenile (Exp. 1 & 2) and adult rats (Exp. 3), expecting stronger behavioral responses in juvenile rats, where 50-kHz calls occur in great numbers [37]. To test the effect of call features, natural 50-kHz calls and artificial sine wave tones (i.e. “calls” without amplitude and frequency modulation) were used (Exp. 2 & 3). In accordance to a bulk of evidence showing that primarily frequency modulated 50-kHz calls are linked to a positive affective state [30], [32], [42], it was expected that they can induce approach behavior. However, it was expected that flat 50-kHz signals might also induce approach behavior, since it was shown that flat calls are predominantly emitted after separation from the cage mate, suggesting that this call serves as a contact call [40], [43].

## Materials and Methods

### Animals and housing

In total, 68 male Wistar rats (HsdCpb:WU, Harlan-Winkelmann, Borchen, Germany) served as subjects. In Exp. 1, 12 juvenile male rats were used, weighing 66.7±2.5 g (range: 52.5–76.5 g; about 25 days of age) on the test day. Twenty juvenile male rats were used in Exp. 2, weighing 80.9±1.5 g (range: 66.0–91.0 g; about 27 days of age) on the test day. Finally, 36 adult male rats were used in Exp. 3, weighing 320.5±6.3 g (range: 273.0–422.0 g; about 12 weeks of age) on the test day. All animals were naïve, except for animals of Exp. 2, which were separated from mother and litter two times for 10 min on postnatal day 11. Animals were housed in groups of 5 (Exp. 2) or 6 (Exp. 1 & 3) on Tapvei peeled aspen bedding (indulab ag, Gams, Switzerland) in a Macrolon type IV cage (size: 378×217×180 mm, plus high stainless steel covers). Lab chow (Altromin, Lage, Germany) and water (0.0004% HCl-solution) were available ad libitum. Animals were housed in an animal room with a 12:12 h light/dark cycle (lights on 7–19 h) where the environmental temperature was maintained between 20–25° Celsius. Prior to testing, all animals were handled for 3 days in a standardized way (5 min each day).

### Experimental setting

Testing was performed on a radial maze of gray plastic with 8 arms (9.8×40.5 cm) extending radially from a central platform (diameter: 24 cm), which was elevated 52 cm above the floor (for details see: [60]). Acoustic stimuli were presented through an ultrasonic speaker (ScanSpeak, Avisoft Bioacoustics, Berlin, Germany) using an external sound card with a sampling rate of 192 kHz (Fire Wire Audio Capture FA-101, Edirol, London, UK) and a portable ultrasonic power amplifier with a frequency range of 1–125 kHz (Avisoft Bioacoustics). The loudspeaker had a frequency range of 1–120 kHz with a relatively flat frequency response (± 12 dB) between 15–80 kHz. It was placed 20 cm away from the end of one arm at a height of 52 cm above the floor. Testing was performed under red light (approximately 11 lux in the center of the maze and between 9 and 12 lux in the arms) in a testing room with no other rats present.

All behavioral tests were conducted between 9–17 h. Prior to each test, behavioral equipment was cleaned using a 0.1 % acetic acid solution followed by drying.

### Acoustic stimuli

**Figure 1:**
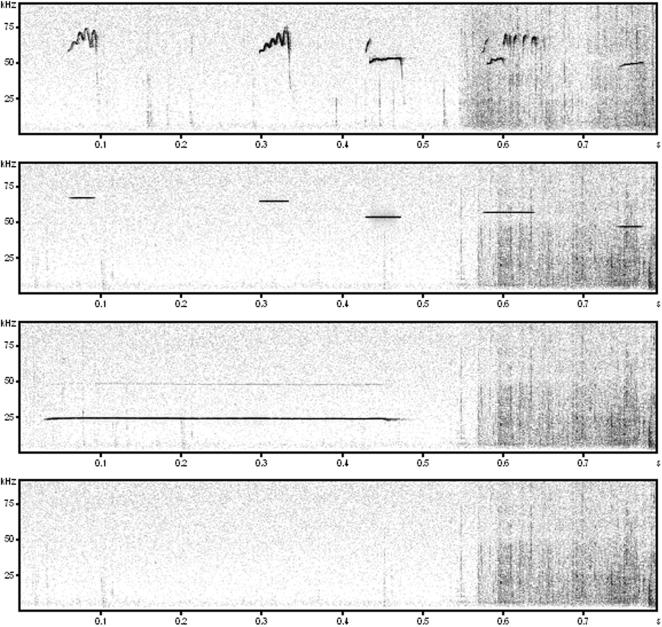
Exemplary spectrograms of the four types of acoustic stimuli presented, namely (from top to down): natural 50-kHz calls, artificial 50-kHz sine wave tones, natural 22-kHz calls, and background noise.

The following four acoustic stimuli were used: 50-kHz calls, 50-kHz sine wave tones, 22-kHz calls, and background noise (see Fig. 1). All stimuli were presented for 1 min with a sampling rate of 192 kHz in 16 bit format. Calls and tones were presented at about 69 dB (measured from a distance of 40 cm), and noise was presented with about 50 dB, which corresponds to the background noise during playback of the other stimuli.

### 50-kHz calls

Throughout playback, 221 natural 50-kHz calls (total calling time: 15.3 s) were presented. The presentation was composed of a sequence of 3.5 s, which was repeated for 1 min, i.e. 17 times, to assure the presentation of a high number of frequency-modulated calls within a relatively short period of time. Each sequence contained 13 calls (total calling time: 0.90 s). Out of these, 10 were frequency-modulated and 3 were flat, and had the following features: call duration 0.07±0.01 s (mean±SEM); peak frequency: 61.24±1.75 kHz; bandwidth: 4.63±1.21 kHz; frequency modulation: 31.68±4.62 kHz. These calls had been recorded from a male Wistar rat during exploration of a cage containing scents from a cage mate (for setting and recording see: [43]).

### 50-kHz tones

50-kHz sine wave tones were generated with the computer software SASLab Pro (version 4.2, Avisoft Bioacoustics) by replacing all calls through sine wave tones. In detail, each given call was replaced by a sine wave tone with identical duration, frequency, amplitude, etc. Thus, the signal had the same temporal patterning and was identical to the 50-kHz call signal with respect to all call features, apart from the fact that the tones were not amplitude and frequency modulated as the natural 50-kHz calls.

### 22-kHz calls

Throughout playback, 29 natural 22-kHz calls (total calling time: 34.25 s) were presented. These calls had the following acoustic parameters: call duration 1.18±0.06 s; peak frequency: 23.61±0.07 kHz; bandwidth: 1.37±0.05 kHz; frequency modulation: 1.90±0.09 kHz. Their presentation was not composed of a repeated sequence, since in case of the long 22-kHz calls potential information, which is contained in temporal patterning is likely lost through sequencing. These calls had been recorded from a male Wistar rat after applications of foot-shocks (for setting and recording see: [10]).

### Noise

Since all three acoustic stimuli presented contained background noise, i.e. sounds, which occur when a rat is exploring an arena with bedding, background noise without calls or tones was presented to control for its possible effects.

### Experimental procedure

A given animal was placed onto the central platform of the radial maze, facing the arm opposite to the loudspeaker. After an initial phase of 15 min where no acoustic stimuli were presented (termed habituation), the rat was exposed to three presentations of acoustic stimuli for 1 min, each followed by an inter-stimulus-interval of 10 min.

Between sub-groups of subjects, different orders of stimulation presentation were used to account for the possible impact of sequence effects. In Exp. 1, background noise, 22-kHz calls and 50-kHz calls were used as acoustic stimuli. They were presented in the following orders: a) background noise, b) 22-kHz calls, c) 50-kHz calls (n = 6 rats), or a) background noise, b) 50-kHz calls, c) 22-kHz calls (n = 6). In Exp. 2 and 3, where background noise, 50-kHz sine wave tones and 50-kHz calls were tested used, they were presented either in the order a) background noise, b) 50-kHz sine wave tones, c) 50-kHz calls (Exp. 2: n = 6; Exp. 3: n = 12), or a) background noise, b) 50-kHz calls, c) 50-kHz sine wave tones (Exp. 2: n = 6; Exp. 3: n = 12), or a) 50-kHz calls, b) 50-kHz sine wave tones, c) background noise (Exp. 3: n = 12), or a) 50-kHz calls, background noise, 50-kHz sine wave tones (Exp. 2: n = 7). One animal was excluded from analysis of Exp. 2 due to incorrect presentation of acoustic stimuli.

We abstained from depicting the order of stimulus presentation in detail, since it had no major qualitative effects on the patterns of result, i.e. behavioral responses towards 22-kHz calls and 50-kHz calls were similar over all positions (Mann-Whitney-U-test for Exp. 1 or Kruskal-Wallis-test for Exp. 2 & 3: all p-values >.100).

### Recording and analysis of animal activity

Behavior was monitored by a video camera (Panasonic WV-BP 330/GE, Hamburg, Germany) from about 150 cm above the maze, which fed into DVD recorder (DVR-3100 S, Pioneer, Willich, Germany).

Behavioral analysis was performed in two ways. A trained observer scored the videos for the time spent on the three arms proximal to or distal from the ultrasonic loudspeaker. Furthermore, the total distance travelled (cm), and the number of arm entries into the three proximal or distal arms, were analyzed using an automated video tracking system (Ethovision, Noldus, Wageningen, The Netherlands). For the automated analysis, input filters were activated to avoid an over-estimation of locomotor activity due to head-movements. In more detail, a minimal distance moved of 8 cm was used for the total distance travelled, whereas a minimal distance moved of 3 cm was used for the arm entries.

### Recording and analysis of ultrasonic vocalization

Playback of acoustic stimuli and potential ultrasonic calls uttered by the rat under testing were monitored by two UltraSoundGate Condenser Microphones (CM 16; Avisoft Bioacoustics) placed 20 cm away from the maze at a height of 55 cm above the floor. One out of these two was placed next to the loudspeaker, i.e. in front of the three proximal arms, whereas the other one was placed vis-à-vis in front of the three distal arms. These microphones were sensitive to frequencies of 15-180 kHz with a flat frequency response (± 6 dB) between 25–140 kHz, and were connected via an Avisoft UltraSoundGate 416 USB Audio device (Avisoft Bioacoustics) to a personal computer, where acoustic data were displayed in real time by Avisoft RECORDER (version 2.7; Avisoft Bioacoustics), and were recorded with a sampling rate of 214,285 Hz in16 bit format.

For acoustical analysis, recordings were transferred to SASLab Pro (version 4.38; Avisoft Bioacoustics) and a fast Fourier transform was conducted (512 FFT-length, 100 % frame, Hamming window and 75 % time window overlap). Correspondingly, the spectrograms were produced at 488 Hz of frequency resolution and 0.512 ms of time resolution. The numbers of 22-kHz calls and 50-kHz calls were counted by experienced observers.

### Statistical analysis

Non-parametric statistics were used, since several data sets were not normally distributed as indicated by the Shapiro-Wilk-test. In more detail, the Friedman-test for repeated measurements was calculated to test whether overt or calling behavior is affected by presentation of acoustic stimuli. When appropriate, the Wilcoxon-test was used subsequently to determine whether overt or calling behavior during presentation of a given acoustic stimulus differ in comparison to other acoustic stimuli, or in comparison to phases without presentations of acoustic stimuli. For the last purpose, overt and calling behavior shown in the three min preceding stimulus application was averaged to eliminate habituation effects. Furthermore, the Wilcoxon-test was used to compare the entries into or the time spent on proximal or distal arms of the radial-maze during a given test period. Finally, Spearman correlation coefficients were calculated to test whether individual responses to different acoustic stimuli were stable and whether overt and calling behaviors were related to each other. The exact p-values of 2-tailed testing were taken as measures of effect.

## Results

### Experiment 1 – juvenile rats

This initial experiment was performed to test whether presentation of ultrasonic calls is effective to modify behavior in juvenile rats. Here, we used 22-kHz calls, for which we expected behavioral inhibition, and natural 50-kHz calls, for which we expected activation and orientation towards the source of stimulation.

### Locomotor activity

**Figure 2:**
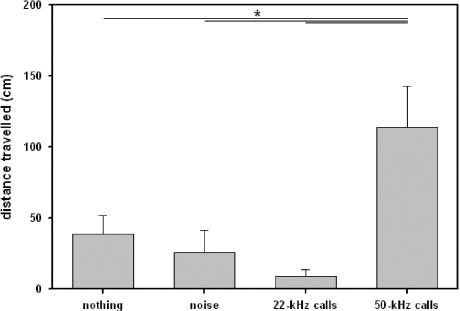
Locomotor activity of juvenile rats in Exp. 1. Bars depict the distance travelled during test phases without acoustic presentation (nothing), presentation of noise (noise), artificial 50-kHz sine wave tones (50-kHz tones), and natural 50-kHz calls (50-kHz calls). Values reflect means±SEM per minute. Animals of all stimulus orders were collapsed, i.e. n = 12. Comparisons with p<.05 are marked with asterisks: *.

Locomotor activity of juvenile rats was affected by presentations of acoustic stimuli (see Fig. 2), since the distance travelled was dependent on a) whether acoustic stimuli were presented or not and b) which type of stimulus was presented. In detail, natural 50-kHz calls caused an increase in the distance travelled in comparison to test periods without presentations (Z = −2.353, p = .016), or to presentation of noise (Z = −2.934, p = .001). In contrast, locomotor activity was reduced when natural 22-kHz calls were presented, indicated by a decrease when compared versus natural 50-kHz calls (Z = −2.746, p = .003), and a trend for a decrease in comparison to test periods without presentations (Z = −1.955, p = .055), but not in comparison to presentation of noise (Z = −.415, p = .734). Finally, no difference in locomotor activity was found between test periods without presentations and background noise (Z = −1.070, p = .322).

### Stimulus-directed locomotor activity

**Figure 3:**
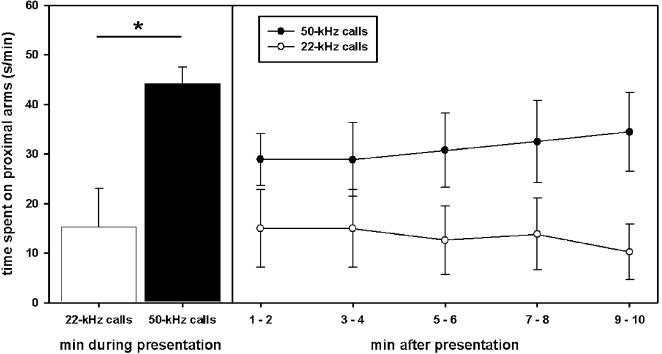
Stimulus-directed locomotor activity of juvenile rats in Exp. 1. The time spent on the proximal arms in front of the loudspeaker is given for playback of natural 22-kHz calls (white bar) and natural 50-kHz calls (black bar) is depicted on the left. On the right, the time spent on the proximal arms in front of the loudspeaker is given for the 10 min after cessation of playback of natural 22-kHz calls (open symbols) and natural 50-kHz calls (filled symbols). Values reflect means±SEM per minute. In both cases, animals of all stimulus orders were collapsed, i.e. n = 12. Comparisons with p<.05 are marked with asterisks: *.

As expected, only natural 50-kHz calls, but not natural 22-kHz calls, induced approach behavior (see Movie S1). Thus, during presentations of 50-kHz calls animals entered the three proximal arms in front of the loudspeaker more often than the three distal ones (Z = −2.456, p = .016) and spent more time in the former (Z = −3.059, p<.001). No preference was observed during playback of noise or natural 22-kHz calls (all p-values >.100). Remarkably, approach behavior during playback of 50-kHz calls was evident despite the fact that the animals showed an a-priori preference for the distal arms, indicated by more entries into distal arms than in proximal ones and the fact that animals spent more time in the distal arms than proximal ones during habituation (Z = −2.185, p = .026 and Z = −2.510, p = .009, respectively) and after cessation of noise (Z = −1.720, p = .084 and Z = −2.134, p = .032, respectively). After playback of 22-kHz calls, no preference was found (all p-values >.100), whereas animals tended to stay longer in proximal arms than in distal ones after presentation of 50-kHz calls (Z = −1.805, p = .076; arm entries: Z = −1.660, p = .110). When comparing the time spent on proximal arms during playback of 22-kHz calls and 50-kHz calls, it was found that animals spent more time on proximal arms during playback of 50-kHz calls (Z = −2.589, p = .007; see Fig. 3). This stimulus-dependent difference was also evident after cessation of acoustic stimuli (Z = −2.040, p = .042), indicating that 50-kHz calls can induce a sustained preference for the source of playback.

### Ultrasonic calling

During testing, 7 out of 12 animals emitted some 50-kHz calls (1.75±0.65, i.e. 0.02±0.01 per min). However, none of them emitted 50-kHz calls during presentation of 50-kHz calls, or 22-kHz calls, and only one animal emitted a single call during presentation of noise, meaning that calls were predominantly emitted during inter-stimulus-intervals (not shown in detail).

22-kHz calls were not observed. However, calls with a similar shape and a long duration up to 900 ms, but an atypical high frequency, were found in one animal, which emitted 15 calls after cessation of presentations of 50-kHz calls (not shown in detail). Remarkably, it emitted also 50-kHz calls.

### Experiment 2 – juvenile rats

Here, we again used juvenile subjects and tested whether behavioral activation and approach might not only be elicited by natural 50-kHz calls, but also by artificial 50-kHz sine wave tones which had the same temporal patterning and were identical to 50-kHz calls with respect to all call features, apart from the fact that the tones were not amplitude and frequency modulated.

### Locomotor activity

**Figure 4:**
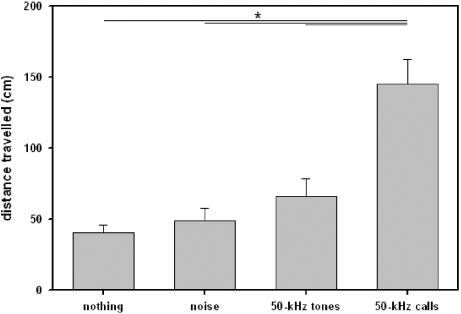
Locomotor activity of juvenile rats in Exp. 2. Bars depict the distance travelled during test phases without acoustic presentation (nothing), presentation of noise (noise), artificial 50-kHz sine wave tones (50-kHz tones), and natural 50-kHz calls (50-kHz calls). Values reflect means±SEM per minute. Animals of all stimulus orders were collapsed, i.e. n = 19. Comparisons with p<.05 are marked with asterisks: *.

In replication of Exp. 1, it was found that 50-kHz calls caused an increase in the distance travelled in comparison to test periods without presentations (Z = −3.662, p<.001), or to presentation of noise (Z = −3.662, p<.001; see Fig. 4). In contrast, playback of 50-kHz tones did not induce locomotor activation, and locomotor activity during presentation of 50-kHz tones was lower as during presentation of 50-kHz calls (Z = −3.340, p<.001; all other p-values >.100). Finally, no difference in locomotor activity was found between test periods without presentations and background noise (Z = −1.046, p = .312).

### Stimulus-directed locomotor activity

**Figure 5:**
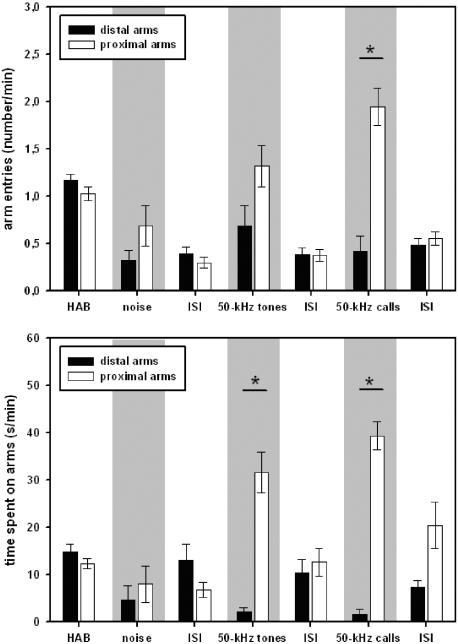
Stimulus-directed locomotor activity of juvenile rats in Exp. 2. The number of entries into the distal (black bars) or proximal (white bars) arms from the loudspeaker is given for habituation (HAB), inter-stimulus-intervals (ISI), and playback of acoustic stimuli, i.e. natural 50-kHz calls (50-kHz calls), artificial 50-kHz sine wave tones (50-kHz tones), and background noise (noise) in the upper figure. The time spent on the distal (black bars) or proximal (white bars) arms from the loudspeaker is given for habituation (HAB), inter-stimulus-intervals (ISI), and playback of acoustic stimuli, i.e. natural 50-kHz calls (50-kHz calls), artificial 50-kHz sine wave tones (50-kHz tones), and background noise (noise) in the bottom figure. Values reflect means±SEM per minute. In both cases, animals of all stimulus orders were collapsed, i.e. n = 19. Comparisons with p<.05 are marked with asterisks: *.

Furthermore, it was found that locomotor activity was stimulus-directed during both, presentation of 50-kHz tones and natural 50-kHz calls (see Fig. 5), since the animals entered the three proximal arms in front of the loudspeaker more often than the distal ones (50-kHz tones: Z = −2.012, p = .055; 50-kHz calls: Z = −3.572, p<.001). Furthermore, they spent more time on the proximal arms than on the distal ones (50-kHz tones: Z = −3.575, p<.001; 50-kHz calls: Z = −3.823, p<.001). Such preferences were not observed during test periods without presentations, or during presentation of noise, except for a trend for a longer time spent on proximal arms relatively to distal ones after the cessation of presentation of 50-kHz calls (Z = −1.811, p = .073; all other p-values >.100).

### Ultrasonic calling

During testing, 10 out of 19 animals emitted 50-kHz calls. However, call rates were very low (1.42±0.58, i.e. 0.03±0.01 per min), and none of them emitted 50-kHz calls during presentation of 50-kHz tones or 50-kHz calls. Solely 1 animal emitted 1 single call during presentation of noise, meaning that 50-kHz calls were predominantly emitted during ISIs (not shown in detail).

22-kHz calls were not observed. However, calls with a similar shape and a long duration up to 900 ms, but an atypical high frequency, were found in some few animals. Throughout the whole testing period, 3 out of 19 animals emitted them (1, 4 and 22 calls). Calls were primarily emitted during the presentations of 50-kHz tones or 50-kHz calls and after cessation of presentations (not shown in detail). Remarkably, 2 out of the 3 animals also emitted 50-kHz calls.

### Experiment 3 – adult animals

In this final experiment, we used the same approach as in Exp.2, and asked whether 50-kHz calls or 50-kHz sine wave tones might also be effective when presented to adult rats.

### Locomotor activity

**Figure 6:**
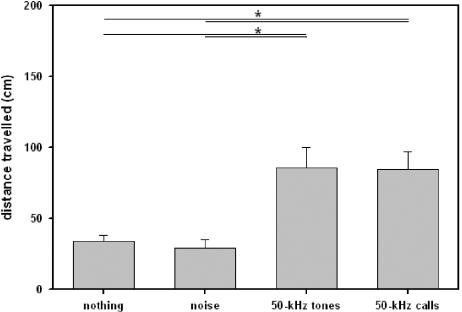
Locomotor activity of adult rats in Exp. 3. Bars depict the distance travelled during test phases without acoustic presentation (nothing), presentation of noise (noise), artificial 50-kHz sine wave tones (50-kHz tones), and natural 50-kHz calls (50-kHz calls). Values reflect means±SEM per minute. Animals of all stimulus orders were collapsed, i.e. n = 36. Comparisons with p<.05 are marked with asterisks: *.

As in juvenile rats, locomotor activity was dependent on on a) whether acoustic stimuli were presented or not and b) which type of stimulus was presented (see Fig. 6). In detail, 50-kHz calls caused an increase in the distance travelled in comparison to test periods without presentations (Z = −.3833, p<.001), or to noise (Z = −3.976, p<.001). Furthermore, a similar increase in the distance travelled was observed when 50-kHz tones were presented (in comparison to periods without presentations: Z = −3.620, p<.001; in comparison to presentation of noise: Z = −3.548, p<.001). Remarkably, the distance travelled did not differ between presentations of 50-kHz tones and 50-kHz calls (Z = −.131, p = .903). Finally, no difference in locomotor activity was found between test periods without presentations and background noise (Z = −1.456, p = .150).

### Stimulus-directed locomotor activity

**Figure 7:**
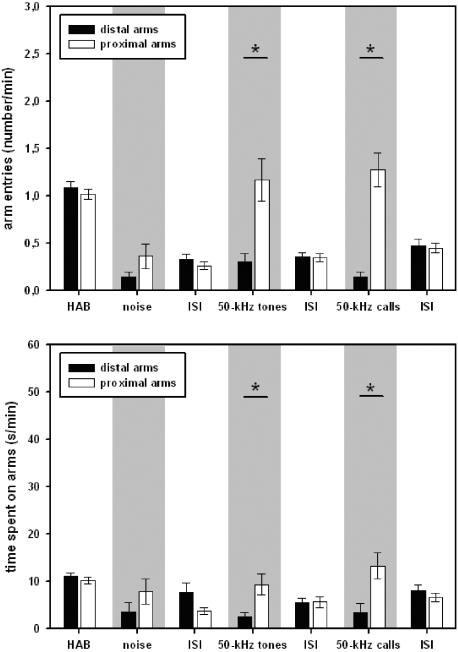
Stimulus-directed locomotor activity of adult rats in Exp. 3. The number of entries into the distal (black bars) or proximal (white bars) arms from the loudspeaker is given for habituation (HAB), inter-stimulus-intervals (ISI), and playback of acoustic stimuli, i.e. natural 50-kHz calls (50-kHz calls), artificial 50-kHz sine wave tones (50-kHz tones), and background noise (noise) in the upper figure. The time spent on the distal (black bars) or proximal (white bars) arms from the loudspeaker is given for habituation (HAB), inter-stimulus-intervals (ISI), and playback of acoustic stimuli, i.e. natural 50-kHz calls (50-kHz calls), artificial 50-kHz sine wave tones (50-kHz tones), and background noise (noise) in the bottom figure. Values reflect means±SEM per minute. In both cases, animals of all stimulus orders were collapsed, i.e. n = 36. Comparisons with p<.05 are marked with asterisks: *.

Locomotor activity was stimulus-directed during presentations of 50-kHz tones and 50-kHz calls (see Fig. 7), since the animals entered the three proximal arms in front of the loudspeaker more often than the three distal ones (Z = −4.110, p = .001 and Z = −3.155, p<.001, respectively). Also, they spent more time on the proximal arms (50-kHz tones: Z = −2.575, p = .008; 50−kHz calls: Z = −2.516, p = .010). Such preferences were not observed during test periods without presentations or presentation of noise (all p-values >.100).

### Ultrasonic calling

**Figure 8:**
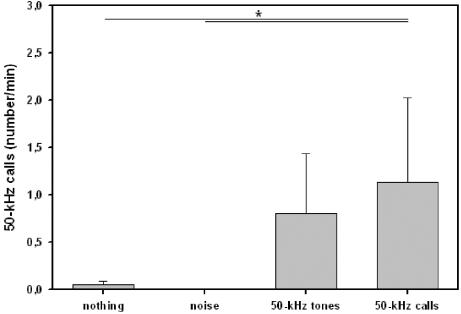
Ultrasonic calling of adult rats in Exp. 3. Bars depict the number of 50-kHz calls emitted by the subject under study during test phases without acoustic presentation (nothing), presentation of noise (noise), artificial 50-kHz sine wave tones (50-kHz tones), and natural 50-kHz calls (50-kHz calls). Values reflect means±SEM per minute. Animals of all stimulus orders were collapsed, i.e. n = 36. Comparisons with p<.05 are marked with asterisks: *

During testing, 26 out of 36 animals emitted 50-kHz calls (5.44±2.49, i.e. 0.11±0.05 per min). Out of these, 8 animals emitted 50-kHz calls during presentation of 50-kHz tones or 50-kHz calls, but none animal emitted 50-kHz calls during presentation of noise. Remarkably, 50-kHz calling was affected by presentations of acoustic stimuli (see Fig. 8). Call emission was higher during presentations of 50-kHz calls than during testing periods without presentation (Z = −2.157, p = .047) or presentation of noise (Z = −2.410, p = .016), whereas call emission during presentations of 50-kHz tones did not differ from any other test period (all p-values >.100), indicating that only playback of 50-kHz calls induced 50-kHz calling. Finally, no difference in calling behavior was found between test periods without presentations and background noise (Z = −1.414, p = .500).

Interestingly, 50-kHz calling was related to activity and approach behavior during presentations of 50-kHz tones and 50-kHz calls. In detail, during presentation of 50-kHz tones the number of 50-kHz calls emitted was positively correlated with the distance travelled (rho = .394, p = .017), the number of entries in proximal arms (rho = .404, p = .014) and the time spent there (rho = .346, p = .039), but not with the number of entries in distal arms (rho = .043, p = .803) and the time spent there (rho = .314, p = .062). During presentations of 50-kHz calls, the number of 50-kHz calls emitted by the subject under study was positively correlated with the distance travelled (rho = .345, p = .039), the number of entries in proximal arms (rho = .386, p = .020) and tended to correlate with the time spent there (rho = .299, p = .076), but no with the number of entries in distal arms (rho = .017, p = .922) and the time spent there (rho = −.147, p = .392) were observed. No correlations between 50-kHz calling and locomotor activity and the direction of locomotor activity were found during habituation (all p-values >.050).

22-kHz calls were very rarely observed. Throughout the whole testing period, only 2 out of 36 animals emitted them. One of them emitted 9 calls after cessation of the presentation of 50-kHz tones, the other one emitted 2 calls after cessation of the presentation of 50-kHz calls (not shown in detail). Remarkably, both animals emitted not only 22-kHz calls, but also 50-kHz calls. Actually, the first one displayed the highest number of 50-kHz calls throughout the whole testing period (90 calls), but also throughout the presentations 50-kHz tones (22 calls) and 50-kHz calls (32 calls).

## Discussion

Our results demonstrate for the first time that 50-kHz calls can induce approach behavior and ultrasonic calling in non-sexual contexts, whereas 22-kHz calls induced a reduction in locomotor activity.

### Playback of 22-kHz calls induce behavioral inhibition

The present findings are in line with several previous ones, which have already shown that 22-kHz calls can activate the fight/flight/freeze system. Dependent on the strain of the receiver, 22-kHz calls can induce behavioral inhibition [48]–[51], or bursts of locomotor running and jumping, which are characteristic of defence behavior [49], [50], [52], [53]. However, it has to be noted that studies using natural 22-kHz calls obtained only a moderate reduction of locomotor activity [48], [51], [59], which is in line with the relatively weak effects of 22-kHz calls found here. From these results, one should not conclude that 22-kHz calls do not provide important signals for the recipient; rather, one should assume that their salience depends on additional features like a given social context [5], or whether they are linked to critical experiences [59].

### Playback of 50-kHz calls can induce activation and approach

Studies on the behavioral effects of 50-kHz calling using playback methods were predominantly conducted in the sexual context. Here, it was found that darting behavior and approaches toward the partner increased in frequency when the female was devocalized, but decreased when tape recorded female ultrasonic calls were presented [56], [57]. With respect to male USV, it was shown that devocalization of male rats resulted in a reduction of female proceptive behavior [61], and playback of 50-kHz calls restored proceptive behavior in oestrus females [23], [25], [27].

The few studies, which were conducted in a non-sexual context, however, obtained very weak or no playback-induced effects on overt behavior. Thus, early studies using artificial ultrasonic stimuli observed Preyer's reflex [54], or a suppression of instrumental bar pressing and bradycardia [55], possibly reflecting an unspecific orienting response. Finally, a recent study by Endres et al. [59], did not observe any change in overt behavioral activity when natural 50-kHz calls were presented in comparison to other acoustic stimuli, like white noise or even 22-kHz calls. Therefore, the present study is the first one, which clearly shows that 50-kHz calls can affect overt and calling behavior in a non-sexual context. In accordance to the hypothesis that 50-kHz calls serve communicative purposes [44], [62], [63], we found that animals increase locomotor activity and approach the source of the stimulus, resembling mothers when searching for their pups in response to isolation-induced pup calls [45], [46], [47].

Furthermore, we showed that playback of 50-kHz calls can elicit ultrasonic calling by the recipient subject, which is in line with findings by White et al. [58] showing that male 50-kHz calls can elevate female calling. Thus, the present findings clearly indicate that the communicative value of 50-kHz calls is not restricted to sexual interactions. Therefore, it can be concluded that differences between sexual and non-sexual contexts are not responsible for the conflicting findings. Possible reasons for the lack of evidence in previous studies might be due to the type of stimulus material and playback technology used in the early playback work [54], [55], or the experimental setting used in the study of Endres et al. [59], who mounted their loudspeaker above the testing arena and not at the side, as done here. Possibly, 50-kHz signals coming from the horizontal axis might provide a more naturalistic signal for the recipient than calls coming from above.

### Frequency modulation is not necessary for eliciting approach behavior

The fact that 50-kHz calls induced approach behavior clearly indicates that these calls were appetitive, which is in line with findings by Burgdorf et al. [32] who showed that rats show instrumental behavior to receive 50-kHz calls. There, frequency-modulated, but not flat 50-kHz calls were effective, whereas the present results demonstrate that 50-kHz signals with and without amplitude and frequency modulation are appetitive, since artificial 50-kHz sine wave tones also induced approach behavior. Despite the fact that natural 50-kHz calls tended to be more efficient in eliciting behavioral changes, amplitude and frequency modulation is apparently not a necessary prerequisite for the appetitive value of 50-kHz calls. Therefore, the present results are more in accordance with the assumption that a whole bundle of call features is responsible for the information, which is conveyed by such calls. We suggest, therefore, a compensatory model for 50-kHz calls, which states that the whole signal information is not lost when a specific call feature is missing, what would be predicted on the basis of the alternative conjunctive model.

Alternatively, one could assume that both, flat and frequency modulated calls, might be appetitive, but that the value of the latter is perhaps higher than that one of flat calls, a difference which is more likely to be detected in tests, like the one used by Burgdorf et al. [32], where the animal can actively chose between playback of different call varieties. Another explanation is that peak frequency rather than frequency-modulation is critical for the appetitive value of 50-kHz calls, since Burgdorf et al. [32] showed that frequency-modulated and flat calls also differ in their peak frequency. In the present study, only the amplitude and frequency modulation of calls was removed, but mean peak frequency remained unchanged, meaning that the 50-kHz sine wave tones used here had a peak frequency, which is typical for frequency-modulated calls. Actually, Brudzynksi [64] has suggested that, apart from call number, peak frequency is involved in coding the quantitative aspect of the sign function of 50-kHz calls, since peak frequency can be modulated by pharmacological agents, like glutamate [65].

### Juvenile rats respond more strongly to 50-kHz calls than adult rats

Furthermore, we found that effects on overt behavior were more pronounced in juvenile rats than in adult rats. This age-related difference is even more impressive, when considering the relatively small number of young animals and the fact that the effect was evident irrespective of whether 22-kHz calls were presented in the same test or not. The difference in approach behavior between juvenile and adult rats is possibly reflecting a decrease in social interest in function of ageing. In fact, a reduced level of gregariousness among older individuals was consistently found in mammals. For instance, in a wide variety of primate species, aging leads to active withdrawal from social interactions and an increase in time spent alone [66]–[68]. Similar changes in function of age were also found in rats and mice. Thus, Salchner et al. [69] were able to show that aged rats spent considerably less time in active social interaction than young rats. Recently, Moles et al. [70] replicated this finding in mice. Interestingly, they did not only observe a decrease in the time spent investigating the partner, but also in the number of USV.

Furthermore, the stronger overt behavioral response in juvenile rats is in accordance with observations that 50-kHz calls occur predominantly in juvenile rats [37]. However, it remains unclear why young animals do not vocalize at all during playback of 50-kHz calls, whereas adult rats displayed ultrasonic calling in response to playback. One point, which might be of relevance in this context, is that the 50-kHz calls presented where emitted by adult rats, and it seems to be possible that call characteristics may convey information about age and status. Apart from these differences between juvenile and adult rats, it was observed that adult rats responded similarly to 50-kHz sine wave tones as to natural 50-kHz calls, whereas the response toward the artificial tones was not as strong as toward the natural calls in young animals. This difference might be due to a reduced acoustic sensitivity and plasticity in adult animals [71].

### 50-kHz ultrasonic calling and social approach

Rats are gregarious. For instance, two rats placed together in a large chamber spend substantially more time together than would be expected by chance, and are more attracted to other rats than to physical objects [72], [73]. Obviously, social approach is crucial for establishing and maintaining relationships among individuals. The present findings indicate that the emission of 50-kHz calls is an important element in the evolvement of social relationships in rats. In fact, 50-kHz calls are typically emitted during social interactions, like reproductive behavior [21], [23], [25]–[28], juvenile play [19], [20] and tickling [36]–[40]. That emission of 50-kHz calls is functional for these behaviors is indicated by studies showing that deafening or devocalizing rats can affect reproductive behavior [23], [25], [27], [28], [56], [61] and reduces rough-and-tumble play [74]. Correspondingly, it was found that animals prefer to spend more time with other animals that vocalize a lot rather than with those that do not [75]. Furthermore, rats emit 50-kHz calls when entering areas where social contact has previously occurred [22], [24], [44], [76], [77]. Remarkably, the present findings nicely fit into earlier studies where it was shown that adult rats emit 50-kHz calls after separation from the cage mate, indicating that such calling serves to (re)establish or keep contact [43]. Similar conclusions can be drawn for mice, where USV was found during mating and social exploration [70], [78]–[81]. Interestingly, Panksepp et al. [80] observed that high-frequency calling in mice is positively correlated with social investigation. Furthermore, Moles and D̀Amato [79] have shown that social investigation and the number of ultrasonic calls can be modulated by manipulating the attractiveness of the test partner. They have suggested, therefore, that ultrasonic calls facilitate proximity between animals, which helps to acquire relevant social information.

The study of social approach in laboratory animals can help to reveal biochemical, genetic and environmental factors underlying neuropsychiatric disorders such as depression, autism and Rett syndrome, since these are characterized, among others, by social deficits and loss of desire to engage in social interactions [82]. Bearing in mind the wealth of evidence implicating 50-kHz calls as a key element of social interactions in rats, it is noteworthy that the measurement of behavioral responses toward playback of 50-kHz calls provides a rather unique opportunity to study the determinants of social interest by using a standardized non-social test, i.e. without confounding effects of a partner. For instance, it is possible to model two core symptoms of the autistic syndrome, namely lack of social interest and communicative deficits [83], [84].

### Conclusion

The present findings clearly show that 50-kHz calls can induce approach behavior and ultrasonic calling in male rats. Thus, the hypothesis that such 50-kHz calls serve for communicative purposes, for example, to (re)establish or to keep contact with conspecifics, is supported.

## Supporting Information

Movie S1 Juvenile rat before and during playback of natural 50-kHz calls.
(27.17 MB MPG)
